# Continuities and discontinuities in pharmaceutical treatment and medication use among older chronically ill patients of Turkish descent in Germany: a qualitative structuring content analysis

**DOI:** 10.3389/fpubh.2024.1358820

**Published:** 2024-08-14

**Authors:** Hürrem Tezcan-Güntekin, Rona Bird, Sema Aslan, Yagmur Kul, Özge Azman, Volkan Aykaç, Beate Klammt, Meryem Aslan, Ilknur Özer-Erdoğdu

**Affiliations:** ^1^Department II: Health and Early Childhood Education, Alice Salomon University of Applied Sciences Berlin, Berlin, Germany; ^2^Berlin School of Public Health, Charité Berlin, Berlin, Germany; ^3^Health Department in the District Office Reinickendorf, Berlin, Germany; ^4^Evangelisches Geriatriezentrum Berlin - Charité Universitätsmedizin Berlin, Berlin, Germany; ^5^Competence Centre for Prevention and Empowerment, Berlin, Germany; ^6^Hochschule Niederrhein, University of Applied Science, Faculty of Nursing Science, Siegen, Germany; ^7^g2 Organisationsentwicklung GmbH, Witten, Germany

**Keywords:** migration, pharmaceutical treatment, medication management, polypharmacy, chronic illness

## Abstract

**Background:**

Polypharmacy occurs frequently among older adults and is associated with an increased risk of falls and medication-related adverse events. In particular, people with a history of migration may receive inappropriate medication due to language barriers or discrimination in healthcare. This study aims to assess the continuities, discontinuities and barriers to drug therapy in older migrants of Turkish descent in Berlin, Germany.

**Methods:**

Eleven problem-centered qualitative interviews with chronically ill older persons of Turkish descent and family caregivers were conducted and analyzed qualitatively by means of structuring content analysis.

**Results:**

The chronically ill participants of Turkish descent predominantly take more than 5 types of medication per day and aim to take them regularly. Discontinuities emerge when medication is forgotten or intentionally omitted due to side effects. Frequent changes in medication and physicians' lack of time are relevant barriers to drug treatment plans. To avoid language barriers and disinterest on the part of professionals, respondents prefer Turkish-speaking physicians.

## Introduction

With increasing age, the likelihood of being affected by multiple chronic illnesses and therefore taking five or more different types of medication concurrently (polypharmacy) increases as well (1–3). People with a history of migration are at a particularly high risk of discontinuous pharmaceutical treatment due to specific barriers (4–7). Against the backdrop of increased global migration, the identification of these barriers has become a key public health concern.

Several studies of European populations consistently indicate that people with a history of migration are more likely to be exposed to certain risk patterns which influence health status and medication use (8–12). Furthermore, a variety of social determinants influence medication management in these European populations. In a sample of first generation migrants (aged > 55) from a variety of ethnic backgrounds, Denktaş et al. (13) identified language proficiency and attitudes toward gender roles as key determinants of medication underutilization. Factors such as language proficiency may affect how the prescription of generic substitutions is perceived, leading to misconceptions regarding the safety of generic drugs (14).

Discontinuity in medication use among older adults with a history of migration may also be exacerbated by transnational living arrangements and cross-border healthcare utilization (15, 16). Sekercan et al. (15, 17) identified dissatisfaction with Dutch primary care as a key motivating factor in cross-border healthcare utilization among persons of non-Dutch descent. In addition to discontinuities in medication use precipitated by cross-border health care utilization, transnational living arrangements may jeopardize medication adherence. Supplies may run out, routines may be altered while traveling or individuals can experience improvements with regards to holistic wellbeing and decide to discontinue medication (16).

Existing literature focusing on patients with a history of migration indicates that medication adherence among migrants depends on their perception of illness, health literacy, and informedness about their specific conditions (18). Factors like language barriers and illness perceptions may make this group particularly vulnerable to discontinuities in pharmaceutical treatment (19). However, in-depth explorations of practices and reflections relating to day-to-day medication management among chronically ill patients and their family caregivers are so far lacking.

From a theoretical perspective, Corbin and Strauss described self-management in the context of chronic illness as the “work of managing illness and daily life” (20). The concept of self-management ascribes agency to people living with chronic illness, conceptualizing the negotiation of everyday life and the healthcare system as an individual coping practice (21). This qualitative study draws on Haslbeck and Schaeffer's (22) approach to self-management, which views the concept as a tool to strengthen patient autonomy and empowerment to take charge of their own wellbeing and collaborate with their healthcare providers on equal terms. This conceptual framework of self-management was employed to illustrate which facilitators and barriers are experienced by patients and caregivers in organizing medication regimes proactively. The study aims to investigate how chronically ill patients of Turkish descent in Germany and their family caregivers experience continuities and discontinuities in pharmaceutical treatment with reference to their attitudes and practices surrounding medication management.

## Materials and methods

### Study design

The study presentend in this article was conducted as one part of a larger research project^1^ examining polypharmacy in people with a migration background in Germany. The project had four components:

1) Eleven problem-centered interviews (23) with chronically ill older people of Turkish descent (8 persons) and family caregivers (3 persons) and a structuring qualitative content analysis (24) investigating medication management and intake practices from the perspectives of patients and family caregivers;
2) Eleven semi-structured expert-interviews (25) and structuring qualitative content analysis (24) investigating prescription and consultation practices in relation to patients with a history of migration from the perspective of healthcare professionals (26);
3) Ten problem-centered interviews (23) and a structuring qualitative content analysis (24) of the impact of protective measures in the context of the Sars-CoV-2 pandemic on the pharmaceutical care of chronically ill people of Turkish descent (27).
4) The development of web applications to improve medication management and interprofessional collaboration.

This article presents findings only from the first part of the study.

A qualitative design was chosen in order to investigate how chronically ill patients and family caregivers experience and reflect on practices of day-to-day medication management with a particular emphasis on continuities and discontinuities. The concept of self-management as practiced by chronically ill individuals coping with their conditions shaped the construction of the interview guide and provided a conceptual framework for data analysis. The decision to employ structuring content analysis within this project was based on the intention to summarize, synthesize, and organize explicit statements made by participants in order to present their thoughts and reflections on medication management.

### Sampling and recruitment

Participants were selected to yield a maximally varied sample with regards to age (while meeting the definition of “older adult”) and educational background. Inclusion criteria for interviews included living with chronic illness, the intake of medications, being over 50 years old and being of Turkish descent. Five districts in Berlin (Neukölln, Kreuzberg/Friedrichshain, Mitte, Spandau and Tempelhof/Schöneberg) were selected for recruiting participants based on their relatively large number of older residents of Turkish descent. Older adults with a history of migration constitute a hard-to-reach population for research participation. Response to general calls for participation that require potential participants to reach out to researchers tends to be low (28), so alongside sending letters to migrant self-help organizations and associations, this study employed recruitment via “gatekeepers,” i.e., key members of communities of Turkish descent who helped interested potential participants to get in touch with the researchers. Recruitment was completed by means of snowball sampling.

### Data collection

Data collection was carried out between December 2019 and February 2020, and was thus completed just as the Sars-CoV-2 pandemic began. Problem-centered interviews were conducted using an interview guide. The problem-centered interview allows for semi-structured qualitative data collection that combines a relatively narrow focus on a specific topic or “problem” with narrative prompts intended to give the participant space to share their subjective experiences (23). The interview guide was developed in collaboration with project partners (DetaMed, Prof. Dr. von Löwis from the Berliner Hochschule für Technik, Töchter und Söhne GmbH) and was pre-tested with two interviewees. The interview guide covered the following topics:

a) Current state of health,
b) Experience of chronic illness,
c) Medication: number and type, intake, prescription/maintenance, continuity,
d) Expectations of medication,
e) Difficulties with medication,
f) Preparedness to use a web application for medication management.

The interview guide was available in Turkish and German, thus allowing participants to choose the language of their interview.

Interviews were conducted in person or by telephone by HTG, YK, IÖW, SA, and ÖA in either Turkish or German and were audio-recorded. The transcription of the interviews was carried out partly by research assistants within the project and partly by a paid transcription service. The interviews that were conducted in Turkish were initially transcribed in Turkish. Qualitative structuring content analysis begins with the identification of relevant text segments and the paraphrasing of those statements. During the paraphrasing of the text segments deemed relevant during this first analytical step, the researchers translated the data to German. Subsequent stages of the analysis were conducted based on the resulting German text segments. Direct quotes that were included in the final analysis were independently translated by two of the researchers in order to meet intercoder-reliability criteria for qualitative research (24).

### Data analysis

The interview data were analyzed using structuring content analysis following Mayring (24). This type of content analysis allows for both deductive and inductive categorisations of data. Based on the research question and the theoretical framework, an initial set of deductive categories was developed. Throughout the analysis, additional categories were developed inductively based on the data. This “bottom-up” process of collaboratively generating new categories based on the data was employed when interview passages were deemed, by researcher consensus, not to fit into any of the previously developed deductive categories. This method ensured that both prior theoretical assumptions and novel content from the interviews were taken into consideration during the analytical process to allow for a comprehensive examination of the material. In order to minimize the impact of subjective judgements, all parts of the analysis were carried out by two researchers independently of each other so that significant discrepancies in interpretation could be discussed reflexively.

The analysis started with the development of a system of categories and subcategories to be deductively applied to the data. Each interview was coded according to the system of categories, while allowing for the inductive development of additional categories when needed. The successive coding of each interview revealed that theoretical saturation had been reached, given the repetition of key themes and eventual decline of novel input in the data. Each coded section of interview text was then paraphrased and subsequently the most essential information from each section was extracted through a second step of “abstraction.”

**Figure 1:**
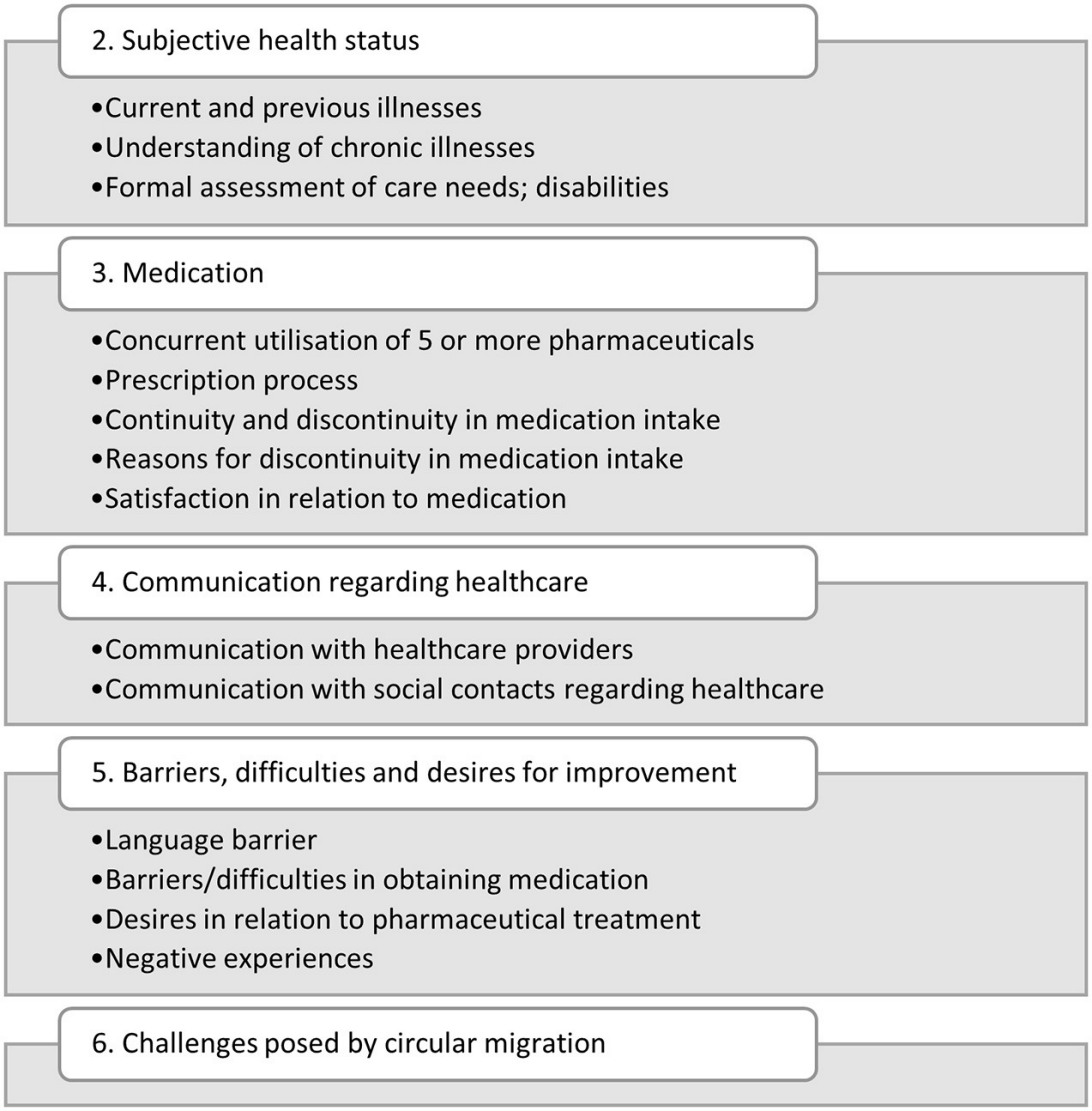
Categories and subcategories 2–6 from data analysis.

A total of 7 categories and 17 sub-categories were developed and applied to the interview data. This article will focus in particular on the categories 2–6. Category 1 relates to the participants' work and migration histories and functioned mainly as a source of contextual information during the analysis. category 7 relates to the potential for using a web-application for medication management and the results will be published separately. Figure 1 shows the categories and subcategories to be presented in this article. Category 6 did not require subcategories.

### Ethical data collection and handling

Before data collection commenced, an ethics vote was granted by the ethics committee of the Alice Salomon University of Applied Sciences in Berlin (Ethics vote 05-2019/24), since chronically ill people and family caregivers constitute a potentially vulnerable population and sensitive health data were collected. Before the data collection process began, all participants were informed in writing (German and Turkish) about the study and the voluntary nature of participation. Informed consent to participate in the study and publication of the results was obtained in writing about 1 week before the interview was conducted. Afterwards a telephone call was offered to answer questions. The interview was recorded by audio and was anonymized during the transcription process. The audio data was deleted after the transcription. Participants could quit the study until transcription. All data was handled in accordance with German data protection regulations.

## Results

### Sample description

A total of 11 interviews were carried out with eight chronically ill patients between 50 and 74 years of age (6 women, 2 men) and three family caregivers between 29 and 42 years of age (one man, two women). Nine of the participants were first-generation migrants, meaning they were born in Turkey and migrated to Germany. Two of the participants were second-generation migrants, meaning the children of migrants who had come to Germany. Years of residence in Germany ranged between 39 and 50 for the first-generation migrants. The age of the interviewees is shown in ranges in the results section to ensure anonymity.

### Subjective health status

Subjective health status was highly varied within the sample and was partly perceived to be negative (5 persons), neutral or average (2 persons), and in some cases good (2 persons). The participants had a variety of illnesses, including diabetes, hypertension, cardiovascular disease, cancer, elevated cholesterol, orthopedic conditions, gynecological conditions, urological conditions, and mental health conditions. Pain was frequently mentioned as a symptom and some participants mentioned having had a large number of surgeries (up to 22) in the past. A good subjective health status was linked to medication use and gratitude for their current medication was expressed by several participants with regards to their health status, even by some participants who perceived their health negatively.

### Number of medications and prescription practice

Interviewees with chronic illnesses frequently took a large number of different medications, with the number of medications ranging between four and seven. The participants gave detailed accounts regarding individual medications and explained what they had been prescribed for. On some occasions, participants appeared to confuse one medication with another and could not describe what they were taken for. Almost all participants complained about side effects and discontinuities, which were described in great detail and traced back to specific events like hospital admissions, surgeries, discharges or prescription changes in the course of discount agreements between health insurance funds and pharmaceutical companies or due to transnational life.

### Perception of chronic illness

Chronic illness were perceived in a variety of ways. Although the inclusion criteria required the presence of chronic illness, the analysis showed that some of the respondents did not perceive their illness as chronic. The following example illustrates that the large number of medications taken is put into perspective:

“*I said that I don't have to take many medications, in the morning I take fasting for thyroid, high blood pressure. Two, three pieces, two for high blood pressure, one for thyroid, then in the evening another one for high blood pressure and one for cholesterol. Then I have another medication for the nerves. If I don't take this one, I cry all the time”* [Interview participant (IP) 2, chronically ill person, 60–69 years old, line 93–97].

### Continuities and discontinuities in the procurement and intake of medication

Medication was prescribed by general practitioners and different specialists. The medication was usually procured continuously from a specific pharmacy in Germany; sometimes the medication was obtained in Turkey. With regard to the continuity of medication intake, it became clear that the relevance of continuous medication intake was understood and achieved by patients. Although patients were aware of the need to take their medication regularly, transnational living posed a challenge, i.e., when participants spent more than 3 months in their home country. These situations can lead to discontinuities and medication changes especially when patients travel with insufficient doses of their medication. As a coping strategy, the patients' German health insurance card was left behind in Germany such that relatives could obtain new prescriptions and send them to Turkey or give them to relatives who were traveling to Turkey for an in-person delivery. Alternatively, the medication was bought in Turkey. However, the change of medication abroad could lead to disruptions in the doctor-patient relationship insofar as the medication prescribed abroad could not be continued in Germany for cost reasons and the patients assumed that they were being denied the medication that is best suited for them.

Discontinuities were also caused by forgetting to take the medication, by leaving out the medication for a few days due to side effects and by intentional omission of the medication. In this case, medication was discontinued without consulting the doctor: “*To be honest, I am careless with the medication for diabetes. Well, not all the time, but now and then I take it. (…) It happens that I forget it because it (the dosage) is low”* (IP4, chronically ill person, 60–69 years old, lines 126–131).

Discontinuities in taking medication also occured during stays in Turkey, when participants reported better subjective health: “*When I'm in Turkey, I don't take any medication at all for a month. (…) I'm supposed to take them every day, but sometimes you just can't do that”* (IP5, chronically ill person, 50–59 years old, lines 131, 213).

### Communication barriers

Communication with doctors and pharmacists was partly rated as positive by patients and family caregivers. Difficulties in the communication with medical or pharmaceutical staff were reported by about half of the interviewees. When such difficulties occurred, they were attributed (a) to doctors' lack of time and professionals' disinterest, or (b) language barriers that were seen as crucial for medication regimens.

(a) Doctors' lack of time and professionals' disinterest

The disinterest is expressed in the following quote: “*When my mom or dad are in pain, it's like they (the doctors) don't really listen to them, or they don't refer them to other doctors for further treatment. Most of the time they say, ‘Take your medication, it will get better.”'* (IP 8, family caregiver, 20–29 years old, lines 85–88). The analysis also shows that, at least in part, doctors' insensitive treatment of patients, lack of time and respondents' lack of education about their disease and the required medication are perceived as barriers: “*He says ‘All right' right away, even before two words have been spoken, ‘I've prescribed the medication, it's ready' he says, ‘You can go home”'* (IP10, chronically ill person, 60–69 years old, lines 281–284). As a result, patients avoid seeking medical care or are dissatisfied with the care they receive.

(b) Language barriers.

Where language barriers existed, the patients tried to overcome this by using Turkish-speaking doctors in Germany. In other cases, there were no language barriers because participants had sufficient knowledge of German.

### Dissatisfaction with side effects and changing medication

While some respondents expressed satisfaction with the effects of their medication, contributing to better subjective health, dissatisfaction was also caused by the large number of pharmaceuticals taken and their side effects. Another reason for dissatisfaction and insecurity were changes in treatment regimens and the prescription of generic drugs. The latter occurred, at least in part, due to the frequently changing rebate contracts between German health insurance providers and pharmaceutical companies. If patients want to continue taking their usual medication, they have to bear the additional costs themselves; alternatively, physicians can specify on their prescription that only a specific medication must be taken.

This quote illustrates the uncertainty that accompanies such changes in medication: “*I'll put it this way, there's no problem with the doctors. They prescribe the usual drugs, but (once I) arrived at the pharmacy, there they say ‘The factory is closed; the name has changed, it's the same,' they say. But for some medicines I insist, ‘I want this, I prefer to wait' I say. There are also the pharmacists that I trust, that I say ‘Okay”'* (IP1, chronically ill person, 70–79 years old, lines 275–278). The analysis shows that trust in pharmacists leads patients to accept changes in medication with confidence.

### Needs and wishes regarding medical care

The older chronically ill people of Turkish descent in this study wanted their medication to be prescribed continuously and without any changes. Moreover, they expressed the need for a critical review of whether their medications are still appropriate and whether they could possibly be discontinued. They would have medications with fewer side effects and a more nuanced patient education regarding their illnesses and the appropriate medications. They further expressed a desire for respectful and friendly treatment by physicians and respect for privacy during examinations.

## Discussion

The findings of our study are in line with existing research that has found the subjective health of people with migration backgrounds to be rather poor, e.g., Razum et al. (6). We also found that the interviewed persons take a high number of medications, i.e., the presence of polypharmacy in our sample of older people of Turkish descent.

For the most part, interviewees made a great effort to take their medication continuously and correctly. Discontinuities in taking medication in the course of transnational lifestyles could be confirmed (15–17, 19) and could be linked to longer stays in participants' country of origin. Medication was also discontinued or changed due to side effects, confirming the results of Håkonsen et al. (14).

While Montesi et al. (4) highlight the risks posed by the underutilisation of primary health care services by migrants with non-communicalble diseases, the present study indicates that older adults of Turkish descent are motivated to seek treatment, but face barriers during medical interactions. These barriers are posed by the lack of time for information provision and patient education as well as the perceived lack of interest expressed by doctors toward our taget group. This may even lead to overuse of health care if patients' needs remain unmet following medical consultations.

The analysis also provided new insights that were not previously available in the state of research: Taking medication is perceived and organized very heterogeneously by people of Turkish descent. However, one common feature can be noted across the sample in this study: participants often took more than five medications and complained about both the large number of medications and their side effects. Language barriers were not identified among all respondents; sometimes they either had sufficient German language skills or they consulted Turkish-speaking doctors and pharmacists. Barriers in the continuous provision of medication included the lack of time and the perceived lack of interest on behalf of professionals. Additionally, the insensitive treatment by doctors was highlighted, resulting in the patients' limited information about the medication and thus affecting adherence.

The aspect of inequality was clearly present in our results, as respondents frequently reported feeling neither well nor comprehensively cared for, while attributing this to their Turkish descent or to language barriers.

Another new finding of our study was that patients' states of knowledge and information about their own illnesses was very heterogeneous. Even when multiple diseases had been diagnosed, patients would sometimes not explicitly attribute these illnesses to themselves. This raises the question of whether and why the medication is then taken in most cases, i.e., a relatively high adherence was recorded. The strong desire for continuous medication and the high adherence identified in this study inspite of limited self-identification with diagnosed illnesses contradicts Shahin et al.'s (18) finding of a negative association between illness identification and medication adherence. Further investigations are needed to clarify why patients who do not explicitly identify as ill may nevertheless adhere well to a medication regime. However, the results showing patients' lack of awareness of and sensitization to chronic illness nevertheless highlight the need for target group-specific and needs-based patient education in health care institutions, which should early on at the stage of diagnosis.

Deploying the conceptual approach of self-management (22), our study has shown that older chronically ill people of Turkish descent develop strategies when dealing with challenges with and to their treatment regimens. They often identify treatment centers where either the doctor or a medical employee speaks Turkish such that they can communicate in their mother tongue. The same applies to pharmacies. Older migrants without language barriers manage their medication very well and perceive fewer barriers to care. The influence of social networks such as adult children who are involved in the care or are available as contact persons seems to be a relevant aspect in the activation of self-management skills with regard to medication. A strong ability to reflect on current treatment by physicians was identified in most cases, as well as an attitude of demanding certain treatments or medications from professionals, indicating strong self-management skills. Other diversity characteristics such as education and language skills seem to have an effect on continuity of medication management.

At the level of pharmaceutical treatment and care, it is necessary to compensate for linguistic and cultural barriers by means of language and cultural mediators. Digital tools are already being developed for this purpose, and their integration into health care practice needs to be examined and evaluated. Increasing the diversity (and diversity sensitivity) of employees with different language skills is an effective solution to this challenge. Even if this is a good approach, it may not be possible to provide all languages in practice It would be more advisable to design practices in such a way that all practices are accessible to people who do not have sufficient command of the language of the target country.

Interprofessional cooperation, e.g., via email or video conference between doctors in Germany and abroad could support transnational care, since retired older people who choose a transnational lifestyle are not isolated cases; rather, a larger population group has chosen this lifestyle.

The aspect of time pressure and possible discrimination by medical staff as a barrier to seeking medical care on a regular basis could be achieved through awareness workshops where a mindful and diversity-sensitive attitude can be practiced and reflected upon. The topic of racism in the healthcare sector has been studied very little in the German-speaking countries, which also indicates a need for further research.

### Future studies should investigate

a) Which forms of discrimination can be identified in the health care of migrants, how these forms of discrimination interact in an intersectional way, and to what extent these intersections affect the provision of medication and regular consultation of doctors, ultimately leading to inequalities in health care provisioning.
b) How the prevalence of polypharmacy among certain migrant groups in Germany relates to the prevalence in the overall population. This could be investigated using quantitative studies based on routine data from health insurance companies.
c) The association between specific experiences of patients in the health care system, illness perception and adherence to medication among older people of Turkish descent in Germany.

Additionally, while Denktaş et al. (13) has shown that language barriers lead to the underuse of health care, it may also be prudent to investigate whether language barriers may also lead to overuse. This requires further studies based on the linking of routine data of the health insurance funds on the prescription of medication with primary data.

## Limitations

Limitations of this study include the fact that the study was conducted in Berlin, an urban area with a very high number of migrants, also amongst doctors and pharmacists. The study therefore could not address self-management strategies in rural areas. An equal representation of men and women in the sample was not achieved. This is primarily due to the well-known difficulties in recruiting older, chronically ill men of Turkish descent for scientific research, which are greater than difficulties in recruiting from the overall population of Turkish descent. We were also unable to reach very old people of Turkish descent as their numbers in Germany are very low due to the timing and age at the time of migration. Moreover, different approaches were sought in the recruitment process; the majority of the interviews were conducted through the networks of the project staff involved acting as gatekeepers, so that a bias could have arisen, even though the networks were very different. Last, because this was a qualitative study that provides insights into individual aspects of people's lives, no generalizable statements can be derived from the results.

## Data availability statement

The original contributions presented in the study are included in the article/supplementary material, further inquiries can be directed to the corresponding author.

## Ethics statement

The studies involving humans were approved by the Alice Salomon University of Applied Science. The studies were conducted in accordance with the local legislation and institutional requirements. Written informed consent for participation in this study was provided by the participants' legal guardians/next of kin. Written informed consent was obtained from the individual(s) and/or the minor(s)' legal guardian/next of kin for the publication of any potentially identifiable images or data included in this article.

## Author contributions

HT-G: Conceptualization, Data curation, Formal analysis, Funding acquisition, Investigation, Methodology, Project administration, Resources, Supervision, Validation, Writing – original draft, Writing – review & editing. RB: Data curation, Formal analysis, Methodology, Writing – original draft, Writing – review & editing. SA: Data curation, Formal analysis, Methodology, Writing – review & editing. YK: Data curation, Formal analysis, Writing – review & editing. ÖA: Data curation, Formal analysis, Methodology, Writing – review & editing. VA: Data curation, Formal analysis, Methodology, Supervision, Writing – original draft, Writing – review & editing. BK: Formal analysis, Methodology, Writing – review & editing. MA: Data curation, Formal analysis, Methodology, Writing – review & editing. IÖ-E: Conceptualization, Data curation, Formal analysis, Investigation, Methodology, Project administration, Supervision, Validation, Writing – original draft, Writing – review & editing.

## Footnotes

^1^“MedikaMig: Vermeidung von Polypharmazie bei Menschen mit Migrationshintergrund”.
